# Comparisons suggest more efforts are required to parameterize wind flow around shrub vegetation elements for predicting aeolian flux

**DOI:** 10.1038/s41598-019-40491-z

**Published:** 2019-03-07

**Authors:** Lin-Tao Fu

**Affiliations:** School of Mechanical Engineering, Chengdu University, Chengdu, 610106 China

## Abstract

Upon interacting with the atmosphere, vegetation could alter the wind distribution and consequently the erodibility of nearby region. The parameterization of wind distribution around vegetation is crucial for the prediction of surface aeolian flux. This paper compared the performances of existing empirical distribution models in the estimation of aeolian flux for shrub vegetation, focusing on distribution pattern and vegetation porosity (main parameter of distribution function). Predicted dust fluxes directly entrained by air flow show weak sensitivity to both distribution pattern and porosity in the case of low vegetation density, which suggests some aspects in dust forecast models might be simplified. However, both distribution pattern and porosity show significant effect on sand saltation transport rate in the lee of vegetation element and, consequently, on the formation and evolution of surface aeolian landforms. The contribution of dust fluxes released in wind increase zone to the total emission by using current parameterizations increases with both the decrease of wind speed and the increase of vegetation density. Nevertheless, the parameterization of wind increase zone needs to be validated and improved by further experimental and numerical investigations.

## Introduction

Vegetation plays a very important role in the change of global climate and the sustainability of ecological system^1–4^. Firstly, as its basic physiological function, vegetation could fix the carbon dioxide in the atmosphere^5^ and hold moisture in the soil^6^. Secondly, as its physical function — being regarded as ground obstacles, it could hinder or reduce the surface aeolian flux through direct or indirect manners^7–11^, which is particularly important for arid or semi-arid regions^1,4^. For example, previous studies suggest that aeolian dust aerosol has direct and semi-direct effect on the climate of Asian arid and semiarid regions^12–14^. Vegetation could directly prevent surface from erosion because of bestrow. It also could form a speed reduction region in the leeward side through interacting with air flow to indirectly decrease the erosion of surface soils^7,8,10,11^. Therefore, it is the key to find a reasonable way to combine the two manners together for quantitatively determining the effects of vegetation on wind erosion^15,16^.

Shear stress partitioning model^1,15,16^ is the most widely used model for qualifying the effects of vegetation on wind erosion. Its core concept is assuming that the dynamical effect of vegetation to an erodible surface is to increase threshold shear velocity (u_*ft_) by absorbing momentum from air flow. Thus, the threshold shear velocity of soil surface covered by vegetation (u_*ft1_) is corporately determined by threshold on bare soil, vegetation basal cover (*C*), lateral cover (*λ*), the ratio of the drag coefficient of roughness element to that of unvegetated soil (*β*) and tuning parameter *m*^15^. The implement of shear stress partitioning model into wind erosion forecast model have been indeed improved the prediction of dust emission^1^. However, there are still uncertainties existing in estimations of the magnitude of dust events^17^ or of the exact location of dust sources^18^. These uncertainties aren’t induced by model precision, but are more likely to originate from the shear stress partitioning model^16,18^. This is because that shear stress partitioning model assumed the same threshold everywhere in vegetated surface (uniform shear stress distribution). In fact, recent experiment^10^ and simulations^11^ all reveal that the shear stress in vegetated surface distributes heterogeneously (non-uniform shear stress distribution). The presence of vegetation reduces aeolian flux by altering (mainly decreasing in the leeward side) the shear stress distribution of local region but not raising the threshold velocity of the whole surface^8^. And a wind erosion model including the distribution of shear stress performs better than that employing stress partitioning model^8,19^. Hence, the parameterization of shear stress distribution around vegetation becomes the key of all issues.

**Figure 1:**
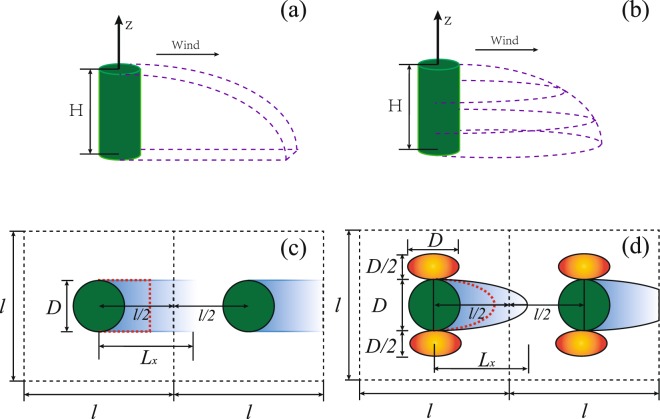
The parameterizations of wind distribution around vegetation element. The zones enclosed by vegetation element and dashed lines in panels (a,b) are proposed wind reduction regions. Panels (c,d) are top view of wind distribution around vegetation. Panel (c): rectangular reduction zone of surface stress distribution for panel (a), panel (d): half-ellipse reduction zone plus full-ellipse increase zone of surface stress distribution for panel (b). In panels (c,d): the red dashed line is the division between the erodible area and non-erodible area for wind reduction region. *D* is the diameter of vegetation element, *l* is the length of the square study region, and *L*_*x*_ is the distance from the central location of vegetation to the location at which the wind speed recovers to the coming wind speed.

The parameterization of shear stress distribution includes two aspects — distribution pattern and distribution function. Typically, the shear velocity is roughly proportional to wind speed at a reference height above surface^10^, hereafter, wind speed distribution is used instead of shear stress distribution. For convenience, a vegetation element is usually simplified as a cylinder^8^. There are three main distribution patterns. The first pattern is the triangle shape proposed by Raupach^7^. He supposed that the wind speed doesn’t vary continuously but is all zero within the zone, while equals the coming wind speed outside the zone. Nevertheless, this assumption seems to be more applicable for solid cylinder cases but less effective for a vegetation element with porous structures^8^. The second one is the rectangular shape proposed by Okin^8^ (Fig. 1a,c). He supposed that the wind speed recovers gradually from the lowest value back to the coming wind speed within the zone. The last one is proposed by Leenders *et al*.^9^ (Fig. 1b,d). Different from the two formers, they proposed that two zones, wind speed reduction zone and increase zone, exist around vegetation. The wind speed reduction zone is assumed to be half-ellipse, while the speed increase zone to be full-ellipse. There are many studies on the wind speed distribution in the leeward side of roughness elements^8–11,20–26^. But systematic and comprehensive description of wind speed around porous vegetation elements is still scare. Here, three typical distribution functions of wind speed reduction for vegetation element are focused. The first function is proposed by Okin^8^ through fitting the experimental data behind a porous windbreak from Bradley and Mulhearn^20^. The second one is proposed by Leenders *et al*.^9^ through fitting their measuring data around shrub vegetation elements (*Hyphaene thebaica* and *Commiphora africana*). But, they only modified the parameters of Hagen’s distribution function^8^, remaining the form unchanged. The last one is recently proposed by Mayaud *et al*.^10^ through fitting their field observed data around three shrub types (*Stipagrostis amabilis*, *Rhigozum trichotomm*, and *Zygophyllum stapfii*). However, they chose the distinguishing form of distribution function in comparison to the first two types. Furthermore, to be more reasonable, Leenders *et al*.^9^ and Mayaud *et al*.^10^ selected both plant height and porosity as the main factors controlling the variation of wind speed in their distribution functions, while Okin^8^ only employed plant height. In contrast, although wind increase zone has also been observed in other studies^10,11^, the description of this zone for porous vegetation was only conducted by Leenders *et al*.^9^.

After a review of literature, it could be found that there is still a lack of studies on comparing the performances of these proposed parameterizations in predicting aeolian flux. Firstly, although the difference in wind speed reduction among different distribution functions was studied^10^, nevertheless, the sensitivity of dust flux release in a vegetated surface to the parameterizations of wind speed distribution (including both pattern and function) isn’t clear yet. Secondly, the importance of the wind increase zone to the estimation of total dust flux in study area isn’t fully understood, and it is also required to evaluate how the sensitivity of increased dust fluxes to parameters that are used to describe the wind distribution in the zone. Thirdly, the sensitivity of saltation flux in the leeward side, which is crucial for the development and evolution of coppice dunes^27,28^, to the parameterizations of wind speed distribution, should be tested. Therefore, this paper aims to investigate these issues.

## Methods

In nature, vegetation elements (or patches) distribute randomly^1,8^. Here, for simplification, the cylinder vegetation elements are assumed to be uniformly distributed in a square area (defined as *l* × *l*)^25,26^, as shown in Fig. 1. The vegetation height is denoted as *H*, and the diameter is denoted as *D*. The basal cover and lateral cover are defined as *C* = *πD*^2^/(4*l*^2^) and *λ* = *DH*/*l*^2^, respectively. The coming wind is supposed to be unidirectional and statistically stable in time and space, which means that no turbulence is considered.

### Patterns and functions of wind speed distribution around vegetation elements

As stated above, two patterns (proposed by Okin^8^ and Leenders *et al*.^9^) are selected here for porous vegetation elements. For rectangular shape pattern (Fig. 1a), the width of the zone equals the diameter of vegetation elements, and the length is the distance, *L*_*x*_, from the central location of vegetation to the location at which the wind speed recovers to the coming wind speed. For the half-ellipse reduction zone (Fig. 1b), the semi-minor axis and semi-major axis are 0.5*D* and *L*_*x*_, respectively. For the full-ellipse increase zone, the semi-minor axis and semi-major axis are 0.25*D* and 0.5*D*, respectively.

Here, the central location of a vegetation element is set as the origin of coordinate, and the wind speed around the element is denoted by $u\ast (x,y)$. The wind distribution function proposed by Okin^8^ for wind reduction zone is shown in Eq. (1), where *C*_*O1*_ = 0.32, *C*_*O2*_ = 4.8, u_*ref_ is the referring shear speed of incoming wind, and *x* is the horizontal coordinate. The distribution function proposed by Leenders *et al*.^9^ for wind reduction zone is expressed in Eq. (2), where ${C}_{Le}=13(0.008-0.17\theta +0.17{\theta }^{1.05})$, *d*_*Le*_ = 1.05$\exp (\,-\,0.5{\theta }^{0.2})$, ${e}_{Le}=2.5(1-0.5\theta )$ and ${f}_{Le}=5-\theta$. *θ* is the vegetation porosity. The distribution function proposed by Mayaud *et al*.^10^ for wind reduction zone is described in Eq. (3). Exactly, ${b}_{M}=1.05\theta +0.1627$, ${u}_{\ast 0}={u}_{\ast ref}(1.46\theta -0.4076)$. The distribution function for wind increase zone is described in Eq. (4). Ø and *C*_*p*_ are the wind-increase factor and area-increase factor, respectively. *x*_*0*_ and *y*_*0*_ are the coordinates of the central location of wind increase zone.

1 $${u}_{\ast }(x,y)/{u}_{\ast ref}={C}_{O1}+(1-{C}_{O1})[1+\exp (-\frac{x}{{C}_{O2}H})]$$

2 $${u}_{\ast }(x,y)/{u}_{\ast ref}=1-\exp [-{C}_{Le}{(\frac{x}{H})}^{2}]+{d}_{Le}\exp [-0.003{(\frac{x}{H}+{e}_{Le})}^{{f}_{Le}}]$$

3 $${u}_{\ast }(x,y)=({u}_{\ast ref}-{u}_{\ast 0})[1-\exp (-\frac{{b}_{M}x}{H})]+{u}_{\ast 0}$$

4 $${u}_{\ast }(x,y)={u}_{\ast ref}\{(1-\O ){[{(\frac{x-{x}_{0}}{0.5D{C}_{p}^{0.5}})}^{2}+{(\frac{y-{y}_{0}}{0.25D{C}_{p}^{0.5}})}^{2}]}^{2}+\O \}$$

### Erosion-related quantities in this paper

Some abbreviations are given at first to help compare performances of published parameterizations of wind speed distributions. In order to understand the differences better, four groups of combinations (pattern + function) are employed. Group 1st is abbreviated as “O2008” which means that both pattern and function are from Okin^8^. Group 2nd is abbreviated as “OM2017” which means that pattern is from Okin^8^ and function is from Mayaud *et al*.^10^. Group 3rd is abbreviated as “L2011” which means that both pattern and function are from Leenders *et al*.^9^. Group 4th is abbreviated as “LM2017” which means that pattern is from Leenders *et al*.^9^ and function is from Mayaud *et al*.^10^.

Five erosion-related quantities are focused in this work. The first quantity is the protecting efficiency (*P*_*r*_) that describes the proportion of the non-erodible area to the total study area. This non-erodible area, within which the wind speed is always lower than threshold value, includes the basal area and the area caused by the sheltering role of vegetation. Mathematically, *A*_o_ = *A*_*e*_ + *A*_*p*_, *P*_*r*_ = *A*_*p*_/*A*_o_, where *A*_0_, *A*_*e*_, *A*_*p*_ are the total study area (*l* × *l*), the erodible area, and the non-erodible area, respectively. The second one is the averaged release rate (*F*_*vt*_) of fluid-entrained PM10 dust in study area. The averaged release rate contains three parts: the release in wind increase zone (*F*_*in*_), the release in wind reduction zone (*F*_*re*_), and the release in normal wind zone (*F*_*nor*_), as shown in Eq. (5). The fluid-entrained PM10 flux could be estimated by the Eq. (6) that is proposed by Zhang *et al*.^29^ on the basis of wind tunnel experimental data. The third one is the surplus PM10 flux (*TF*) defined as the differential of fluxes between considering and without considering the increase of wind speed in wind increase zone, as shown in Eq. (7). The fourth one is the proportion *P*_*in*_ of the *TF* versus the total flux that doesn’t consider the effect of wind increase, as shown in Eq. (8). The last one is the reduction of transport rate (*Q*_*r*_) defined as the differential of rates between considering and without considering the decrease of wind speed within wind decrease zone, as shown in Eq. (9). It’s employed to statistically evaluate the integrated reduction level of transport rate caused by the sheltering role of a single vegetation element. The saltation transport rate *q*_*x*_ is estimated by Eq. (10) ^30^, where u_*it_ is the impact entrainment threshold, *g* the gravitational acceleration and *ρ*_*a*_ the air density. *q*_*x*,0_ tells the estimated rate by using the referring shear speed, while *q*_*x,re*_ by using the reduced shear speed.

5 $${F}_{vt}=[\mathop{\iint }\limits_{{A}_{in}}{F}_{in}dxdy+\mathop{\iint }\limits_{{A}_{re}}{F}_{re}dxdy+\mathop{\iint }\limits_{{A}_{nor}}{F}_{nor}dxdy]/{A}_{0}$$

6 $$F=9.88{u}_{\ast }^{10}(1-{u}_{\ast ft}/{u}_{\ast })\,{u}_{\ast }\ge {u}_{\ast ft}$$

7 $$TF=\mathop{\iint }\limits_{{A}_{in}}({F}_{in}-{F}_{nor})dxdy$$

8 $${P}_{in}=\mathop{\iint }\limits_{{A}_{in}}({F}_{in}-{F}_{nor})dxdy/({F}_{vt}{A}_{0}-\mathop{\iint }\limits_{{A}_{in}}({F}_{in}-{F}_{nor})dxdy)$$

9 $${Q}_{r}=\mathop{\iint }\limits_{{A}_{re}}({q}_{x,0}-{q}_{x,re})dxdy/(\pi {D}^{2}/4)$$

10 $${q}_{x}=2.61{\rho }_{a}({u}_{\ast }^{2}-{u}_{\ast it}^{2})({u}_{\ast }+{u}_{\ast it})/g\,{u}_{\ast }\ge {u}_{\ast it}$$

### Other settings

Shrub is the common vegetation type in arid and semiarid regions^4^. Considering that the proposed parameterizations of wind speed distributions are based on the data measured around shrub vegetation, the diameter and the height of shrub vegetation are employed here. According to the measurements^9,10^, *D* and *H* are taken as 1 m and 0.5 m, respectively. The vegetation porosity *θ* ranges from 0.3 to 0.7^10^. The lateral cover *λ* ranges from 0.03125 to 0.125. The mean diameter of soil particles is assumed to be 0.25 mm and the impact threshold u_*it_ is taken as 0.2 m/s^3,31^. Because of the randomness and complexity of soil surface, the fluid threshold for PM10 is taken as 0.3 m/s^29,32^. The value of the incoming wind speed *U*_*z*_ is set to be the data measured at 10 m above ground. In the cases of predicting the release of PM10, the aerodynamic surface roughness *z*_0_ and the referring shear speed u_*ref_ are determined by Eq. 11^8^, where *κ* is the von Karman’s constant and taken as 0.41. In the cases of predicting sand transport rate behind a single vegetation element, *z*_*0*_ is estimated as *D*/15^33^. The wind-increase factor Ø ranges from 1.08 to 1.16 and the area-increase factor *C*_*p*_ ranges from 0.8 to 1.2. For comparison, shear stress partitioning model is also employed to predict PM10 (taking u_*ft1_ instead of u_*ft_ in Eq. 6), and thus, *β* and *m* are taken as 202^1^ and 0.16^15^, respectively. *g* and *ρ*_*a*_ are 9.8 m/s^2^ and 1.225 kg/m^3^, respectively.

11 $${z}_{0}=\{\begin{array}{c}(0.48\lambda +0.001)H\,\lambda  < 0.11\,\\ 0.0538H\,\,\,\,\,\,\,\lambda \ge 0.11\end{array}{u}_{\ast ref}=\kappa {U}_{z}/ln(z/{z}_{0})$$

## Results

**Figure 2:**
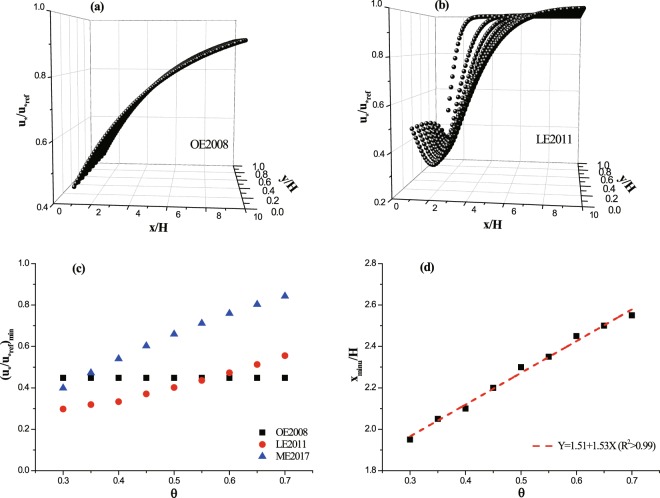
Comparisons of parameterized wind distribution in the lee of vegetation element. Panels (a,b) are spatial distributions of dimensionless leeward wind speed proposed by Okin^8^ (denoted as OE2008) and Leenders *et al*.^9^ (denoted as LE2011), respectively. Panel (c): minimum values of u_*_/u_*ref_ versus porosity. Panel (d): leeward location of minimum u_*_/u_*ref_ versus porosity for LE2011. ME2017 indicates the wind distribution function is proposed by Mayaud *et al*.^10^. x_minu_ is the leeward location of minimum u_*_/u_*ref_.

At the beginning, the proposed parameterizations of leeward wind distribution are compared (Fig. 2). For convenience, OE2008, LE2011 and ME2017 are employed to represent the wind distribution functions proposed by Okin^8^ (Eq. 1), Leenders *et al*.^9^ (Eq. 2) and Mayaud *et al*.^10^ (Eq. 3), respectively. Figures 2a,b show the spatial patterns and wind speed distributions proposed by Okin^8^ and Leenders *et al*.^10^, respectively. Considering the symmetry of wind speed to *x* axis in *xy* plane, only wind speeds in y > 0 region are plotted. Because these three proposed functions are based on experiments in which no measurements were conducted within vegetation, so, only wind values at *x/H* > 1 are considered here. Different from OE2008 where dimensionless wind speed (u_*_/u_*ref_) increases monotonously with *x/H*, u_*_/u_*ref_ in LE2011 decreases first and then increases after reaching a minimum value in *x* direction. Therefore, the minimum values of u_*_/u_*ref_ between the three functions are compared (Fig. 2c). OE2008 only includes the effect of vegetation height, and the minimum values of u_*_/u_*ref_ thus don’t change with vegetation porosity. The minimum values of u_*_/u_*ref_ in both LE2011 and ME2017 increase with porosity. However, the values as well as their increasing rate with porosity in ME2017 are larger than those in LE2011. The minimum values of u_*_/u_*ref_ occur at the leeward edge of vegetation in OE2008 and ME2017; while, the location of minimum u_*_/u_*ref_ increases linearly with porosity.

**Figure 3:**
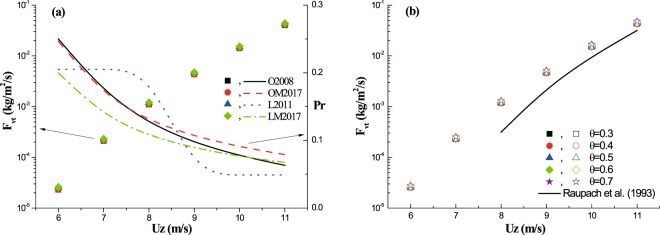
The estimation of erosion-related quantities with the increase of wind speed within the whole study area. Left vertical axes in panels (a,b) are PM10 flux (*F*_*vt*_), and right vertical axis in panel (a) is the protecting efficiency (*P*_*r*_). In panel (a): the porosity *θ* = 0.4, basal cover *C* = 0.04909, and lateral cover *λ* = 0.03125. In panel (b): the black solid line tells the results from shear stress partitioning model proposed by Raupach *et al*.^15^; the solid and open scatters are results from L2011 and LM2017, respectively.

A vegetation element could extend its effective protecting area, where no wind erosion occurs, by the sheltering effect. But, the degree of extension depends on wind speed, as shown in Fig. 3a. With the increase of incoming wind speed, the protecting efficiency *P*_*r*_ decreases towards basal cover over all groups. Nevertheless, there are still some differences. At the lowest wind speed, the *P*_*r*_ of rectangular distribution pattern (O2008 and OM2017) reaches around 1.2 times higher than that of half-ellipse pattern (L2011 and LM2017). Different from LM2017 that *P*_*r*_ decreases gradually, a sharp decrease of *P*_*r*_ occurs between wind speed 8~9 m/s in L2011. This difference should be caused by the wind speed distribution function. In contrast, the averaged release rate of PM10 (*F*_*vt*_) increases with the incoming wind speed, but shows very weak sensitivity to both distribution pattern and function (solid scatters in Fig. 3a). It also reveals weak sensitivity to vegetation porosity (scatters in Fig. 3b), no matter what the wind speed is. Besides, Fig. 3b shows two remarkable differences in prediction of dust release between by considering the distribution of wind speed (scatters) and by without considering the distribution of wind speed (i.e., using shear stress partitioning model) (solid line). Firstly, the estimated PM10 fluxes by considering wind speed distribution are generally higher than those by without considering wind speed distribution. Secondly, it can be seen that, there is no emission of PM10 when the wind speed is below 8 m/s for without considering wind speed distribution, while the emission of PM10 still happens by considering wind speed distribution.

**Figure 4:**
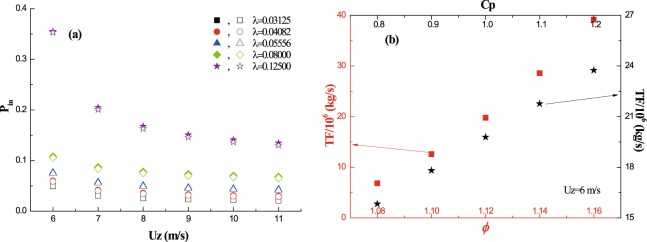
The estimation of erosion-related quantities in wind speed increase zone. Panel (a): The relative proportion (*P*_*in*_) of *TF* with the increase of wind speed; the solid and open scatters are results from L2011 and LM2017, respectively. Panel (b): *TF* versus both wind-increase factor *ϕ* (bottom horizontal axis) and area-increase factor *C*_*p*_ (top horizontal axis).

It reveals that, at a fixed lateral cover, the relative proportion (*P*_*in*_) of *TF* reduces with the increase of wind speed for both distribution functions (Fig. 4a). And, the difference in the relative proportion between two functions is negligible. Also, as shown in the figure, the lateral cover affects the relative proportion significantly. At a fixed wind speed, the relative proportion increases with lateral cover. For example, the value of *P*_*r*_ in the case of *λ* = 0.125 is 6~7 times larger than that in the case of *λ* = 0.03125, which seems to be invariant with wind speed. The tests of the sensitivity of *TF* to main parameters (*ϕ* and *C*_*p*_) are shown in Fig. 4b. It reveals that, the *TF* increases with both *ϕ* and *C*_*p*_. However, in comparison to area-increase factor *C*_*p*_, *TF* shows higher sensitivity to wind-increase factor *ϕ*. Exactly, curve fit of data (R^2^ > 0.98) suggests *TF* increases with *C*_*p*_ linearly, while increases with *ϕ* exponentially. Thus, the ratio of the largest value to the lowest value for *TF* is about 1.5 within the given range of *C*_*p*_, but that for *TF* is higher than 5 within the given range of *ϕ*.

**Figure 5:**
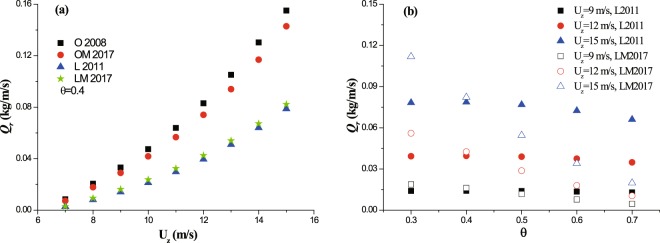
The reduction of transport rate (*Qr*) versus both wind speed (Panel (a)) and porosity (Panel (b)) after a single vegetation element.

The sand transport rate focused here is calculated on the basis of the hypothesis that only one vegetation element exists in the whole flat land surface. The wind distribution function proposed by Okin^8^ shows no strict constraint on the end point of sheltering zone. The calculating results in Fig. S1 in supporting information suggest that the reduction of transport rate (*Qr*) reaches saturation when the leeward distance is about 20 *H*. So, the sheltering length is approximately set to be 20 *H* when the transport rates are calculated by using Okin’s distribution function. As shown in Fig. 5a, *Qr* increases with wind speed over all groups. The transport rates calculated by rectangular pattern (O2008 and OM2017) are far higher than those by half-ellipse pattern (L2008 and LM2017). Under the same pattern, the difference in transport rates owing to distribution functions is small. Figure 5b shows the change of *Qr* with vegetation porosity by using two different wind speed distribution functions. Although *Qr* decreases with vegetation porosity (because the leeward wind speed would increase with vegetation porosity according to proposed parameterizations), the response of transport rate to porosity is both wind-dependent and function-dependent. It could be found that, the increase of incoming wind speed could enhance the variation of *Qr* caused by vegetation porosity (by comparing the square scatters with the triangle scatters). Generally, *Qr* calculated by the distribution function of Leenders *et al*.^9^ is less sensitive than that by the distribution function of Mayaud *et al*.^10^. For example, in the case of *U*_*z*_ = 15 m/s, *Qr* decreases about 15% (referring to the maximum value) for L2011 within the given range of porosity, but decreases about 80% for LM2017 within the same range.

## Discussion and Conclusion

Prediction of dust events is not only important for human being’s health and safety but also for our understanding of the climate change of our planet^3,12–14^. It is thus crucial for us to accurately estimate the dust load in vegetated land surface, particularly in arid and semi-arid region^3,4^. As the most important driving factor for aeolian dust emission, three selected parameterizations (OE2008, LE2011 and ME2017) of leeward wind speeds were compared firstly. Significant differences in the lowest wind speed as well as the corresponding location exist among the three parameterizations, which should originate from the difference in vegetation canopy shape^8–10^. However, the total release rate of dust flux, which is directly entrained by air flow, shows weak sensitivity to both distribution pattern and porosity in the case of low vegetation density (low coverage). Although the contribution of dust release in wind increase zone is considered, the proportion of dust release in wind increase zone accounts for about 5% of total dust emission in the case of low vegetation density or high wind speed, which wouldn’t alter the weak sensitivity. This thus suggests that some aspects in dust forecast models might be simplified. For instance, it seems that the difference in canopy shape owing to vegetation types as well as their growth stages couldn’t need to be considered in low vegetation density locations. Nevertheless, it doesn’t mean that the dust release predicted by shear stress partitioning model (uniform distribution of shear stress^7,15^) would agree with that by the method here (non-uniform distribution of shear stress^8,19^), in the case of low density. Calculated results reveal that dust release could occur in much lower wind speed by considering non-uniform distribution of shear stress than that by uniform distribution of shear stress. Therefore, employment of uniform distribution of shear stress is one of possible reasons that why current dust forecast models yield uncertainties in the location and magnitude of dust events^16^.

The importance of wind increase zone in the lateral side of vegetation elements is tested in this work. Under the settings proposed by Leenders *et al*.^9^, the contribution of wind increase zone in total dust release gradually increases with both the increase of vegetation density and the decrease of incoming wind speed. When the average wind speed over the whole study area is below fluid threshold (no matter this is caused by either the low incoming wind speed or the high vegetation cover), the increase of wind speed around vegetation could make the local speed raise up to a value above fluid threshold and consequently lead to dust emission. So, the increase of wind speed around vegetation element may be another reason that causes the uncertainties by current dust forecast models. Calculating results also reveal the sensitivity of increased dust flux to the two main parameters (wind-increase factor and area-increase factor). It is thus important and valuable to make sure whether the proposal on the parameterization of wind increase zone is reasonable or not. Mayaud *et al*.^10^ also detected the wind increase in the lateral side, but they didn’t quantitatively describe the extent and magnitude of wind increase zone. Their study further suggested that the ratio of height to diameter (or width) and the porosity could affect the acceleration of wind speed. Hence, it is required more data (including experimental, numerical and theoretical data) to confirm the parameterization of wind increase zone by including more related physical factors.

The change of sand transport rate is the key to the formation and development of coppice (or nebkha) dunes in the lee of vegetation element^27,28^. Although total dust release rate is weakly sensitive to both distribution pattern and function, the reduction of transport rate is sensitive to them. For a single element, the half-ellipse pattern seems to be more reasonable than rectangular pattern, according to recent measurement^10^ and numerical simulation^26^. The difference in sensitivity of reduction of transport rate to vegetation porosity, which may be caused by the different canopy shape between LE2011^9^ and ME2011^10^, suggests that more efforts are required to carefully determine the effect of porosity on the recovery of leeward wind speed.

Furthermore, two additional points needs to be noticed. Firstly, the possible change of distribution pattern or function due to the increase of vegetation coverage should be investigated. It should be reminded that these results obtained in this work may be only applicable in low density cases. The interaction among vegetation elements could greatly change the flow pattern (e.g., skimming flow) in moderate or high density cases^34^, consequently, the distribution pattern or function couldn’t be suitable any more. Besides, experiments^35,36^ suggest the canopy shape has great effect on vegetation drag coefficient; and numerical simulation^11^ shows the effect of canopy shape on leeward wind speed distribution. The wind speed at the lowest grid in dust forecast models might thus be estimated inaccurately with the increase of vegetation density because of the difference in canopy shape, which could also be able to rise up the uncertainties in prediction of dust events. Also, the sensitivity of total fluid-entrained dust flux to both wind distribution pattern and porosity in moderate or high vegetation density may be different from that in low vegetation density as shown above. Secondly, the effect of height on the sheltering role of vegetation should be constrained. All distribution functions introduced here didn’t show any constraint on the effect of height, indicating that the sheltering role of vegetation is proportional to vegetation height. In another word, if the height is infinite, the sheltering role would be infinite. This is not the truth. For a fixed diameter (or width), the effect of height on the sheltering role is limited. Raupach^7^ proposed an empirical expression to conceptually describe the variation of sheltering with the ratio of height to width. However, the parameters in the expression are difficult to obtain. Sadique *et al*.^26^ recently proposed a simple way (dividing the effect at the location of roughness height) to limit the effect of element height, based on the numerical data of rectangular solid roughness elements. Thus, their try affords us lesson and could be altered to apply in the study of porous vegetation element.

In conclusion, this paper compared the performances of existing empirical distribution models in predicting aeolian flux for shrub vegetation, focusing on distribution pattern and vegetation porosity (main parameter of distribution function). Canopy shape shows significant influence on wind distribution around vegetation element. Results reveal that the predicted protecting areas would vary, to some extent, among different models; however, the predicted dust fluxes directly entrained by air flow are weakly sensitive to both distribution pattern and porosity in the case of low vegetation density, regardless of canopy shape. The predicted dust fluxes in wind increase zone mightn’t be overlooked, particularly in the cases of low incoming wind and high density conditions. The dust fluxes in wind increase zone show the sensitivity to both area-increase factor and wind-increase factor, but are likely to be more sensitive to the latter. The predicted sand saltation transport rate in the lee of a vegetation element is sensitive to both distribution pattern and porosity. The difference in the reduction of transport rate owing to distribution pattern of wind reduction zone increases with wind speed, while the variation of transport rate with porosity is function-dependent. Rectangular pattern produces more reduction of transport rate than half-ellipse pattern. The *Qr* predicted by the distribution function of Mayaud *et al*.^10^ is more sensitive to vegetation porosity than that by the distribution function of Leenders *et al*.^9^. This difference in sensitivity comes directly from the different leeward wind speed variations. These variations are controlled by the role of porosity in distribution functions that is affected by canopy shape (or shrub type). It thus implies the necessity of considering the canopy shape (or shrub type) in further understanding of the form and evolution of leeward surface landforms. These results shown above suggest that, we need more experimental measurements or numerical simulations to confirm or improve the wind distribution model around shrub vegetation, in order to exactly determine aeolian flux in the presence of vegetation.

## Supplementary information

Supporting information

## Data Availability

All data generated or analysed during this study are included in this published article (and its Supplementary Information files).
