# Supplementary material for: Comparisons suggest more efforts are required to parameterize wind flow around shrub vegetation elements for predicting aeolian flux
Source: Sci Rep. 2019 Mar 7;9:3841. doi: 10.1038/s41598-019-40491-z (PMC6405760; doi:10.1038/s41598-019-40491-z)
Supplement: Supplementary file 1 — Supporting information [file 41598_2019_40491_MOESM1_ESM.docx]

***Manuscript:***

**Comparisons suggest more efforts are required to parameterize wind flow around shrub vegetation elements for predicting aeolian flux**

Lin-Tao Fu^1,*^

^1^School of Mechanical Engineering, Chengdu University, Chengdu 610106, China

Corresponding author:

Lin-Tao Fu^*^

Email address: fultofficial@hotmail.com

**Supporting information**

Fig. S1. The reduced transport rate (*Qr*) versus dimension leeward distance *x/H*.
